# Design of Nonbituminous Binders for Road Application Using Vegetable Resources

**DOI:** 10.1021/acssuschemeng.5c01625

**Published:** 2025-04-28

**Authors:** Rocio Vidal, Rodrigo Álvarez-Barajas, Antonio A. Cuadri, María J. Martín-Alfonso, Pedro Partal

**Affiliations:** Pro2TecS-Chemical Process and Product Technology Research Centre, Department of Chemical Engineering, 16743 Universidad de Huelva, ETSI. Campus de “El Carmen”, 21071 Huelva, Spain

**Keywords:** biobinder, asphalt, rheology, rosin, waste cooking oil, cellulosic pulp, product engineering

## Abstract

This work proposes novel biobinders as a more sustainable alternative to traditional bituminous products. They should be able to fully replace petroleum bitumen as binders of aggregates in road asphalts. Colophony resin ester (R), waste cooking oil (O), and cellulosic pulp (CP) were used as the main components of biobinders. Furthermore, the addition of a small amount of a reactive isocyanate-terminated prepolymer (MDI) is required. Binder formulation and processing were assessed by a comprehensive rheological, microstructural, and technological characterization to understand the role of each component in the final material properties. Rosin was a structuring agent, oil acted as a plasticizer, and the cellulosic pulp increased the material's softening point. MDI played a key role as a compatibilizer via urethane/amide linkages between the isocyanate groups of MDI and the OH/COOH groups present in the other three components. As a result, a biobinder formulation was proposed to replace bituminous binders, which was composed of 27.22 wt % oil, 67.76 wt % rosin, 2.02 wt % cellulosic pulp, and 3 wt % MDI. They should be added following the order R > O > MDI > CP and processed at 150 °C. Finally, its potential as an asphalt binder was evaluated according to European and American standards accepted for commercial bituminous products.

## Graphical abstract

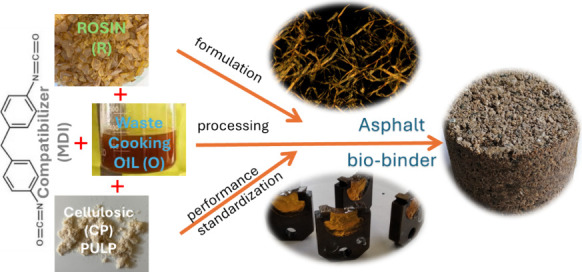

## Introduction

There is a growing interest in adopting sustainable practices in the construction sector that may have significant environmental impacts, including resource depletion and waste generation.^1,2^ This fact is promoting the shift toward environmentally sustainable construction methods that address managerial, strategic, and operational complexities.^3^ In this context, by embracing a circularity approach, waste materials from a wide range of sectors, including food, agriculture, forestry, and heavy industry, have become significant sources of bitumen modifiers.^4,5^

In particular, agriculture and forest exploitation are significant contributors to waste/byproducts production, generating substantial quantities of biomass, waste materials, and different byproducts.^6^ Typically, biomass ends up burned for energy or used as a fuel source in factories and mills.^7^ As an alternative, wood resin, cellulose, or waste plastic have proved to have the greatest value in binder modification.^8–11^ Although these materials have been extensively used as bitumen modifiers or bitumen extenders, few attempts have been made to replace bitumen completely as the binder of asphalt mixes or roofing formulations.^12–14^ For both applications, the challenge lies in selecting bio and waste materials that can effectively substitute bitumen while maintaining or enhancing its characteristics.^8,15,16^

As a result, a reduced number of publications and patents have successfully formulated nonbituminous binders capable of fully replacing crude oil bitumen in road and roofing materials.^17^ Such binders can be broadly classified into two categories: (A) the so-called synthetic binders, which are mainly characterized by their nonbiobased nature^18,19^ ; or, alternatively, (B) biobased binders, in which vegetable oils and/or wood byproducts are blended with various polymers, exhibiting comparable physical properties and low temperature performance to those of conventional 50/70 bitumen.^8,20^

On these grounds, this work aims to develop binders formulated with at least 97% biobased raw materials, which are expected to fully replace bitumen in road applications. These biobinders are mostly formulated by a pine resin ester with the role of a structuring agent, a waste bio-oil as a rosin plasticizer, and a cellulose-rich rheology modifier.

The use of wastes or biobased oils adds environmental value to construction materials and can impart improved impermeability and flexural strength to binders.^21,22^ While different crops have been proposed for the production of nonedible bio-oils to be used in specific technological applications, the increasing volume of waste generated by the food industry has rendered oil waste a more sustainable valorization pathway.^23,24^ Among them, waste cooking oil has emerged as a readily available source of nonedible oils, as further processing is not needed.^25^

The second component of these biobinders is a rosin ester derived from pine resin. Colophony resin has historically been used in the waterproofing of wooden ships.^26^ It is mainly composed of resin acids, especially abietic acid, and appears as a yellowish-to-brownish, semitransparent, and brittle substance. As an alternative to colophony resins, rosin esters are used to improve the softening point and thermophysical, mechanical, and functional properties of the final products. Thus, these materials find application in the manufacture of curing agents, elastomers, surfactants, coatings, adhesives, and hardeners.^26–28^

Finally, cellulose has been widely used as a bitumen modifier or to prevent bitumen drainage during hauling of the Stone Mastic Asphalt (SMA) mixes.^29,30^ Instead, the use of cellulose-rich fibers as polymeric modifiers of biobased binders has not been proposed elsewhere due to their low compatibility with the other binder components (e.g., rosin ester and waste oil). To that end, this work addressed the use of a small amount of an isocyanate-terminated reactive prepolymer, which is expected to play a compatibilizing role among the three binder components.

As a result, this work proposes novel biobinders with improved characteristics as a more sustainable alternative to traditional bituminous binders for road applications. With that aim, this work addresses a comprehensive rheological, microstructural, and technological characterization of the formulated binders, which evaluates the performance of these materials as asphalt binders and seeks to understand the role of each component in the final material properties. The resulting binders are expected to provide a greener and more environmentally friendly alternative to traditional construction materials formulated with petroleum derivatives.

## Experimental Section

### Materials

Formulated binders consisted of a rosin ester (R) supplied by Luresa, S.A. (Spain); a waste cooking oil (O), mainly used for food frying and supplied by BIOLIA, S.A. (Spain); a cellulosic Kraft pulp (CP); and reactive isocyanate-based prepolymer, 4,4′-methylenediphenyl diisocyanate (MDI), provided by Merck (Spain). Rosin was a stabilized pentaerythritol ester of colophony resin with a melting point of 108 °C and 12 mg of KOH/g of acid number. Waste oil came from a blend of vegetable oils commonly used for deep-frying food. It was collected from restaurants, homes, and other catering activities. Waste cooking oil is a mixture of mono-, di-, and triglycerides of fatty acids, together with triacylglycerols, phospholipids, sterol esters, lipids, free fatty acids, triglyceride dimers and oligomers, oxidized triglyceride monomers (with hydroxy, keto, epoxy, and other groups), etc. Its typical composition includes a high content of oleic and linoleic fatty acids (around 80–90 wt %), total polar compounds in the range 9–37 wt %, an acidity around 1.3–7.5, 5–12 wt % oligomer and dimers, 1–5 wt % free fatty acid, and 7–14% polymers.^31^ They result from a series of reactions, including hydrolysis, polymerization, and oxidation, which occur during the frying of food at high temperatures. Its viscosity and density at 100 °C were 1 mPa·s and 0.8773 kg/m^3^, respectively. Cellulosic pulp (CP) was obtained in our laboratory from *Populus* × *Euroamericana clone AF2*, by means of a Kraft process, containing 77.14% cellulose, 15.95% hemicellulose, and 1.72% Klason lignin. Resultant fibers presented a length-weighted average of 1.090 mm, 25.35 μm thickness, and 0.116 mg/m coarseness. Furthermore, thermogravimetric analysis revealed that CP presented around 5% moisture (Figure 1A). Before its addition to binder composition, CP was crushed in an IKA MF10 inline mill using a cutting-grinding head with a 1.5 mm hole-size sieve (Figure 1B). This process resulted in the fibrous microstructure shown in Figure[Fig fig1]C.

**Figure 1:**
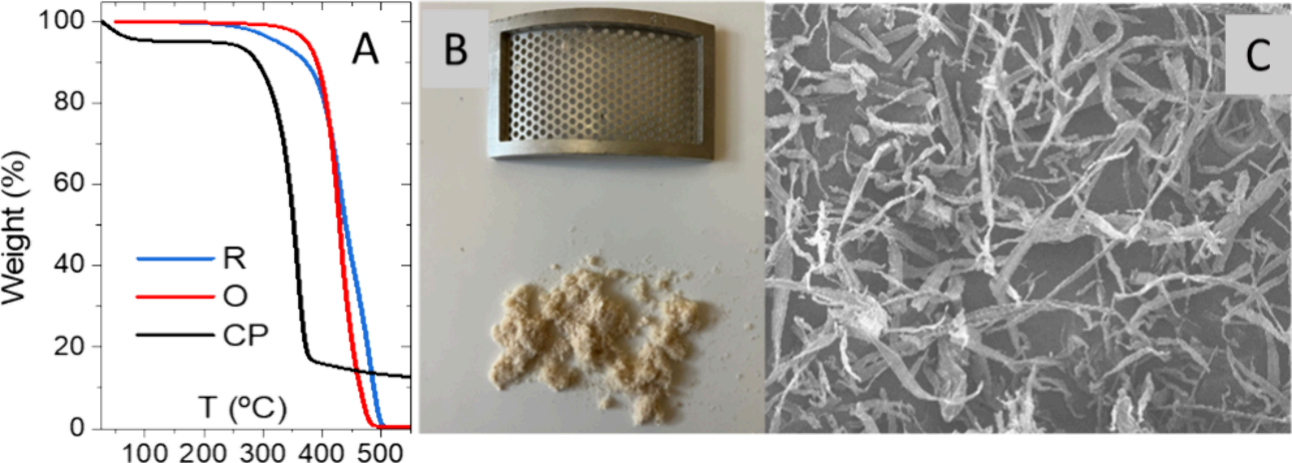
(A) Thermogravimetrical analysis of rosin (R), waste cooking oil, and cellulosic pulp (CP); (B) grinded cellulosic pulp by a sieve with a 1.5 mm hole size; and (C) resultant biopolymer microstructure observed under SEM (100k magnification).

Additionally, commercial neat bitumen B50/70 and SBS polymer modified bitumen PMB 45/80–60 were used as reference materials. The neat bitumen had 56 dmm penetration, 53.5 °C softening point, and a SARAs composition of 4.8% saturates, 52.3% aromatics, 24.5% resins, and 18.4% asphaltenes. PMB had 55.4 dmm penetration, 62 °C softening point, and 85% elastic recovery, being classified as PG76-28 by the ASSHTO MP320.

### Formulations and Processing

Samples were formulated by dissolving rosin and waste oil at a fixed ratio of R/O = 2.5 to obtain a homogeneous blend. This blend resulted in a penetration and softening point in the range of a soft bitumen 160/220, which was considered as a suitable “base” binder for further modifications. Polymers were then added at concentrations ranging from 1 to 5% CP and 0 to 3% MDI. Processing was carried out in a Silverson L5 Laboratory mixer for 120 min at 150°C and 2100–4100 rpm agitation speed. The temperature was chosen to be around 150°C in order to obtain fast reaction kinetics but to avoid thermal decomposition or release of the binder compounds.^32^ The selection was based on the TGAs shown in Figure 1A, and the boiling point of the polymeric MDI (170°C). The total processing time was similar to that used for the preparation of SBS modified bitumen. Batches of 400 g of binder were prepared using a general-purpose disintegrating head with a square-hole high-shear screen. Additionally, for the sake of comparison, products were scaled up to 4000 g batches using a Duplex mixing assembly. Table 1 summarizes the compositions for all systems prepared and their processing and storage conditions.

**Table 1:** Composition, Processing, and Storage Conditions of Rosin-Oil Blends (R/O−) and Binders (B−)

system	composition				addition order	processing *T* = 150 °C			storage *T* = 150 °C
	R (%)	O (%)	CP (%)	MDI (%)		*t*_R/O_ (min)	*t*_CP_ (min)	*t*_MDI_ (min)	*t*_St_ (h)
R/O–1% CP	70.6	28.4	1	0	R–O–CP	30	60	0	0
R/O–2% CP	69.83	28.05	2.12	0	R–O–CP	30	60	0	0
R/O–3% CP	69	27.75	3.25	0	R–O–CP	30	60	0	0
R/O–4% CP	68.5	27.53	3.97	0	R–O–CP	30	60	0	0
R/O–5% CP	67.68	27.2	5.12	0	R–O–CP	30	60	0	0
B–2% CP–2% MDI–ord1	68.48	27.5	2.07	1.95	R–O–CP–MDI	30	60	30	0
B–3% CP–2% MDI–ord1	67.65	27.2	3.18	1.97	R–O–CP–MDI	30	60	30	0
B–2% CP–3% MDI–ord1	67.76	27.22	2.02	3.00	R–O–CP–MDI	30	60	30	0
B–2% CP–3% MDI–ord1-St	67.76	27.22	2.02	3.00	R–O–CP–MDI	30	60	30	24
B–2% CP–3% MDI–ord2	67.76	27.22	2.02	3.00	R–O–MDI–CP	30	30	60	0
B–2% CP–3% MDI–ord2-St	67.76	27.22	2.02	3.00	R–O–MDI–CP	30	30	60	24
B–2% CP–3% MDI–ord2–4 kg	67.75	27.22	2.03	3.00	R–O–MDI–CP	30	30	60	0
B–2% CP–3% MDI–ord2–4 kg-St	67.75	27.22	2.03	3.00	R–O–MDI–CP	30	30	60	24

The order of the addition of binder compounds was also evaluated (Table 1). Order 1 (referred to as R–O–CP–MDI or “ord 1”) consisted of mixing rosin and oil for 30 min (*t*_R/O_); followed by CP addition and mixing for another 60 min (*t*_CP_), and then MDI addition (*t*_MDI_) while keeping mixing up to complete 120 min processing. Alternatively, Order 2 (referred to as R-O-MDI-CP or “ord 2”) consisted of 30 min of rosin-oil mixing; followed by MDI addition and mixing for another 60 min; and, finally, CP addition and mixing up to 120 min maximum processing time. Finally, selected binders were subjected to high-temperature storage for 24 h in an oven at 150 °C (samples labeled as “St”).

### Material Testing

Binder technological characterization involved the following European Standards: EN1426, EN1427, EN12607, EN 12595 and EN14770. Binder consistency was determined by penetration tests, as stated by the European standard EN 1426, measuring the depth that a needle penetrates a sample of binder at 25 °C. Material softening points (also referred to as the ring-and-ball softening temperature) are measured at the temperature at which a steel ball deforms the binder contained within a metal ring under the specified testing conditions outlined in EN 1427. These tests were conducted on fresh and aged samples. Binder aging was carried out in an oven M81-B0161 from Controls (Italy) following an RTFOT methodology (EN 12607), which simulates binder aging during its mixing with the aggregate. During the Rolling Thing Film Oven Test (RTFOT), a film of hot bituminous binder in motion is oxidized with a constant supply of air at a defined temperature and time. Aging tests were performed at a temperature of 150 °C, which was eventually used for the manufacture of asphalt mixes. Furthermore, aiming to study binder behavior after long-term aging, RTFOT-aged samples were artificially aged in a pressure aging vessel (PAV) (Prentex model 9300, USA) at 100 °C and pressurized with air to 2.10 MPa, according to EN 14769 or AASHTO R 28.

Rheological characterization was conducted in a SmartPave 102e rheometer (Anton Paar, Austria) and consisted of frequency sweep tests in oscillatory shear (EN14770) and steady viscous flow measurements (EN 12595). Oscillatory frequency sweep tests, from 100 to 0.01 rad/s, were performed between −20 and 80 °C, combining two plate–plate geometries (8 and 25 mm diameters with 2 mm and 1 mm gap, respectively). All oscillatory shear measurements were carried out at strain values within the linear viscoelastic region (LVR). Viscous flow tests at 135 °C were carried out using a coaxial cylinder geometry (with bob and cup diameters, respectively, *D_i_* = 26.667 mm and *D*_o_ = 28.937 mm) and ranging shear rates between 0.1 and 100 s^–1^.

Sample chemical characterization was conducted by FTIR analysis in a JASCO instrument in an FT/IR 4200 type A (Jasco, Canada). Thin KBr discs or pellets were prepared in a hydraulic press at 5 bar for approximately 3 min with a sample/potassium bromide ratio of 1/10 wt %. Spectra were collected with a resolution of 4 cm^–1^ from 450 to 4000 cm^–1^. Finally, binder microstructure was observed by optical microscopy, using a microscope, an Olympus BX51 (Japan) with a digital camera, an Olympus C5050Z. Polarized light micrographs were taken at room temperature.

## Results and Discussion

### Material Performance vs Formulation

Initially, potential biobinders were formulated by increasing the concentration of cellulosic pulp (CP) added to a rosin-waste oil blend with a fixed R/O ratio of 2.5, previously selected. The resultant three-component system consisted of a homogeneous continuous phase of rosin softened by the dissolved waste oil, the R/O blend, and a disperse phase of the cellulosic biopolymer, hardly compatible with the R/O blend. As may be seen in Figure 2A, the addition of CP to the R/O blend leads to stiffer binders with higher softening points and penetration values tending to be lower. However, this effect is more evident above 3 wt % CP, likely because below this concentration, CP is not stable in the R/O continuous phase and tends to settle, affecting the measurements.

**Figure 2:**
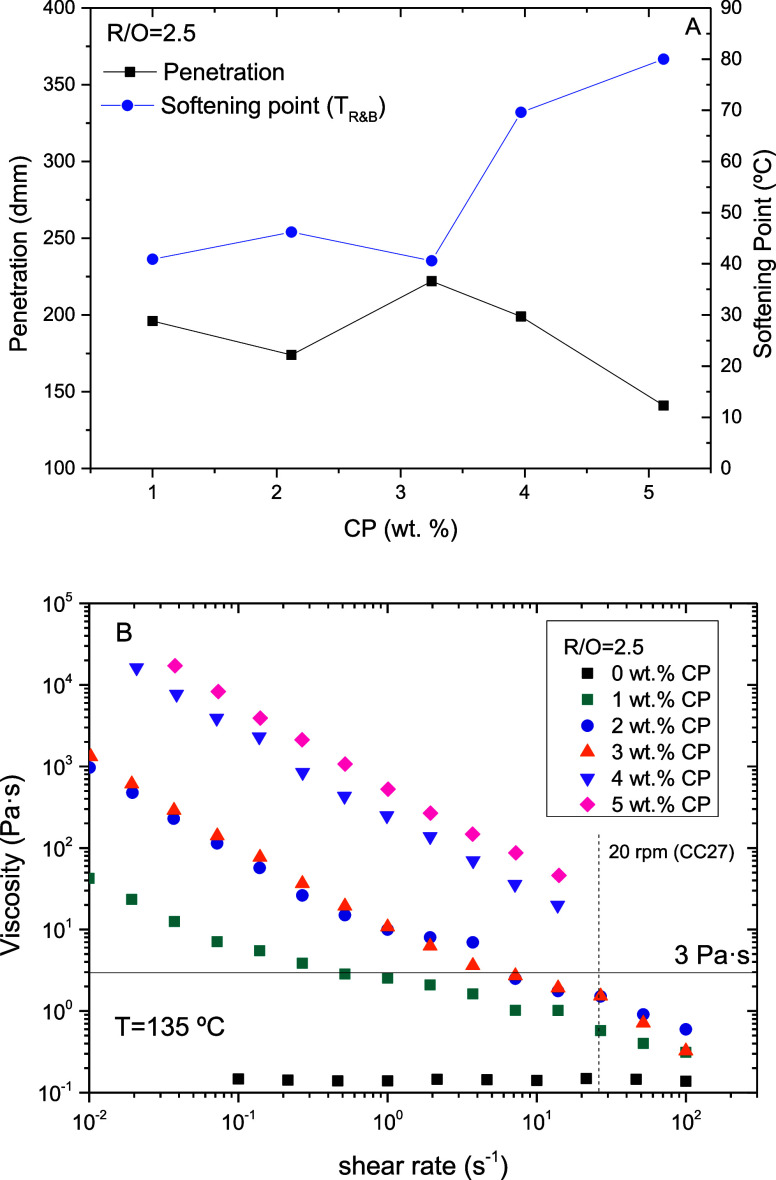
(A) Ring and ball softening points (*T*_R&B_) and penetrations values; and (B) viscous behavior at 135 °C of binders formulated with a rosin/waste oil ratio R/O = 2.5 and different concentrations of cellulosic pulp (CP).

Likewise, CP addition also induces a change in the binder's viscous behavior at temperatures above the rosin/oil melting point (Figure 2B). Unlike the Newtonian R/O blend (sample labeled as 0 wt % CP), systems containing CP increase their viscosity and behave as non-Newtonian (shear-thinning) fluids with viscosities that decrease with the shear rate. This behavior suggests that CP remains undissolved and dispersed within the R/O continuous phase after mixing, forming a polymeric network that deforms and, likely, breaks down under shear forces (responsible for the shear thinning behavior).

It is worth noting that an increase in CP up to 4–5 wt % results in binders with softening points between 70 and 80 °C. These values are in the range of polymer-modified bitumens (PMB) typically demanded in road construction, according to the European Standards (e.g., EN 14023). However, pulp addition does not have the same effect on binder hardness, with high penetration values ranging roughly from 200 to 125 dmm for the most concentrated systems (i.e., too soft to be used in asphalt mixes) (Figure 2A). Moreover, CP remarkably increases the binder viscosity (Figure 2B). This fact may limit the applicability of the most concentrated systems if recommendations gathered in the American standard AASHTO MP320 are taken into consideration. This standard specifies a limiting viscosity of 3 Pa s at 135 °C measured at a 20 rpm rotation speed (AASHTO T316), or at a shear rate around 25–27 s^–1^, calculated for a 17.46 mm diameter spindle rotating in an 18.8 mm diameter cup. Below this viscosity, the binder would be suitable for the handling, lay-down, and compaction of the asphalt mix. As may be seen in Figure 2B, only those systems formulated with less than 3 wt % CP would fulfill this specification at shear rates above 2 s^–1^.

Accordingly, only those ternary systems formulated with concentrations below 3 wt % CP would be suitable as asphalt binders. However, as mentioned above, they are too soft and have a low softening point to be used for this type of application. Aiming to overcome this issue, an isocyanate-based (NCO) reactive prepolymer (MDI) was added to ternary systems with a CP concentration below 3 wt % (Table 1). Terminated-NCO reactive groups are expected to react with active hydrogens available in the waste oil, rosin, and cellulosic polymer, as hydroxyl (−OH) and carboxylic (−COOH) groups. Cellulose and hemicellulose present in CP are known to have a high density of available hydroxyl groups.^33^ Similarly, rosin esters are a source of hydroxyl and carboxyl groups, the latter dependent on their acid index.^34^ Likewise, recycled oil is a mixture of various compounds, including mono-, di-, and triglycerides of fatty acids, as well as triacylglycerols, phospholipids, sterol esters, lipids, free fatty acids, triglyceride dimers and oligomers, and oxidized triglyceride monomers (with hydroxy, keto, epoxy, and other groups). These compounds result from hydrolysis, polymerization, and oxidation reactions induced by the high temperatures during food frying.^35^ They are also an excellent source of carboxyl groups.

Accordingly, NCO would link the above reactive groups mainly through the reactions shown in Scheme 1.^36,37^

**Scheme 1:**
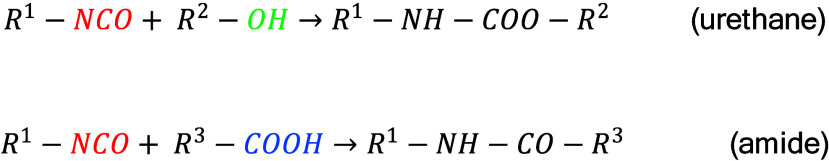
Expected Reactions between Isocyanate group and Hydroxyl (−OH) and Carboxylic (−COOH) Groups Available in the Waste Oil, Rosin, and Cellulosic Polymer

Moreover, by using a polymeric diisocyanate as the MDI, such reactions are expected to promote compatibilization among the three components and a further modification in the system. This assumption is confirmed in Table 2 that shows how the addition of 2–3 wt % MDI to systems containing 2 and 3 wt % CP reduces penetration and increases softening points of binders prepared according to the addition order 1. Among them, it is worth noting the results obtained for the formulations B–3% CP–2% MDI and B–2% CP–3% MDI, with softening points above 60 °C and penetration values in the range 50/70 (Table 2).

**Table 2:** Softening Points (*T*_R&B_) and Penetration Values of Binders Containing MDI as Functions of Their Compositions and Order of Addition

system	CP (wt %)	MDI (wt %)	addition order	penetration (dmm)	*T*_R&B_ (°C)
B–2% CP–2% MDI–ord1	2.07	1.95	R–O–CP–MDI	83	56.5
B–3% CP–2% MDI–ord1	3.18	1.97	R–O–CP–MDI	60	77
B–2% CP–3% MDI–ord1	2.02	3.00	R–O–CP–MDI	48	60
B–2% CP–3% MDI–ord1-St	2.02	3.00	R–O–CP–MDI	70	56.5
B–2% CP–3% MDI–ord2	2.02	3.00	R–O–MDI–CP	60	60.9
B–2% CP–3% MDI–ord2-St	2.02	3.00	R–O–MDI–CP	63	58.2
B–2% CP–3% MDI–ord2–4 kg	2.03	3.00	R–O–MDI–CP	59	57.2
B–2% CP–3% MDI–ord2–4 kg-St	2.03	3.00	R–O–MDI–CP	54	56.9

Even though the system with the best performance seems to be B-3%CP-2%MDI (Table 2), this binder is characterized by high viscosity at 135 °C (Figure 3). As may be seen, for a 2 wt % MDI, the increase in CP concentration up to 3 wt % induces a viscosity rise of about 1 order of magnitude, exhibiting both binders similar shear-thinning behavior (i.e., similar log–log slope of the viscosity vs shear rate curve). Conversely, the addition of 3 wt % MDI to a 2 wt % CP appears to have a compatibilizing effect, reducing the shear-thinning character of the binder, while keeping its viscosity below the limiting value of 3 Pa s at shear rates higher than 25 s^–1^.

**Figure 3:**
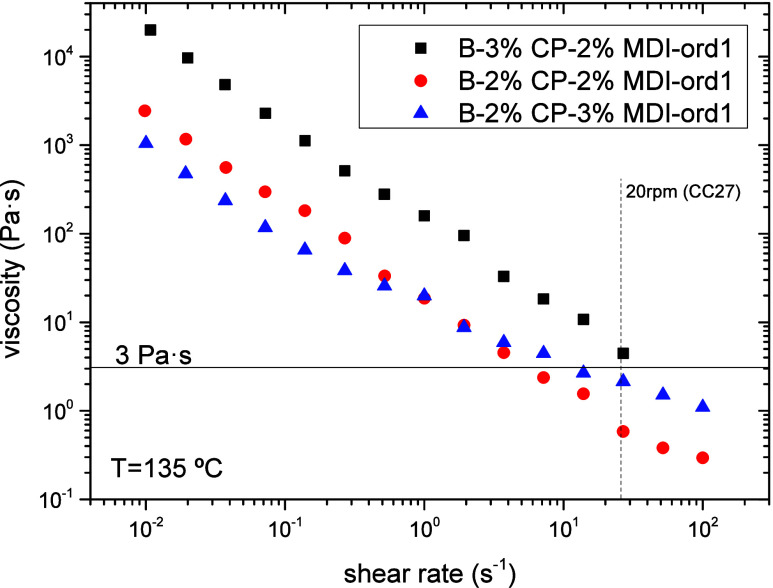
Viscous behavior of biobinders formulated with MDI and processed following the addition order 1 (R > O > CP > MDI).

If both the performance and viscous behavior of all formulated binders are considered as a whole, binder B–2% CP–3% MDI–ord1 would best balance both properties, being selected for further studies.

### Binder Processing and Storage

As previously seen, reactive modification involving NCO and OH/COOH groups may be a promising approach to obtain binders with a suitable performance. However, previous studies on bitumen modification by isocyanates pointed out that resultant PMBs may undergo further modification through different curing mechanisms (e.g., during binder storage in tanks at high temperature).^38^

Aiming to study the effect of an eventual hot storage on these binders, above selected formulation B–2% CP–3% MDI–ord1 was stored at the processing temperature, 150 °C, for 24 h. As may be seen in Table 2, after such storage conditions, the resulting binder (referred to as B–2% CP–3% MDI–ord1-St) partially loses the modification initially achieved, becoming softer (with a lower softening point and, particularly, higher penetration). In the same way, hot storage also reduces the binder viscosity at 135 °C (Figure 4A).

**Figure 4:**
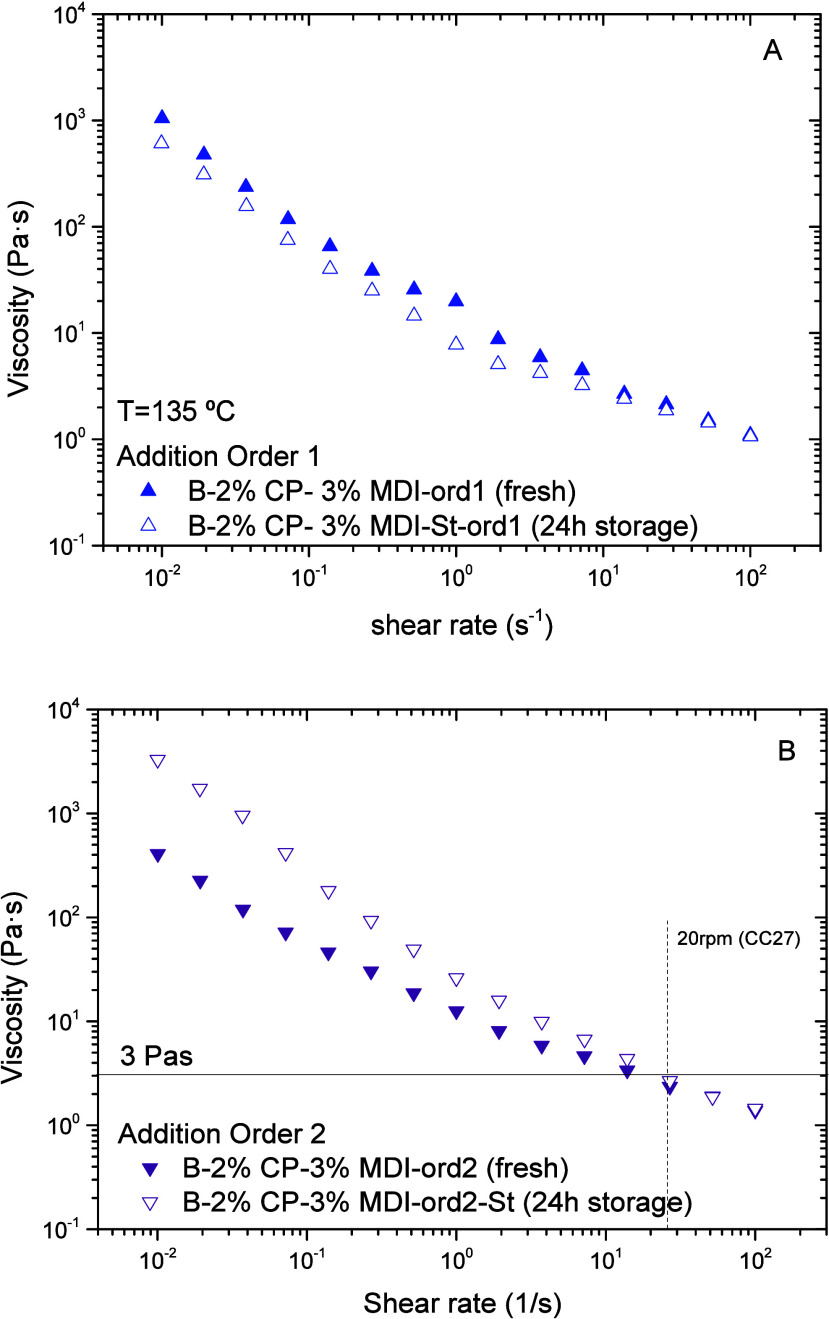
Effect of hot storage at 150 °C for 24 h on binder viscosity as a function of component addition order followed during processing: (A) order 1 (R > O > CP > MDI); and (B) order 2 (R > O > MDI > CP).

Interestingly, the above-observed binder softening can be prevented by changing the order in which the components are added to R > O > MDI > CP (referred to as oder2 or “ord2” hereinafter). As a result, the softening point and, particularly, penetration values remain close to their initial values after hot storage (Table 2). Similarly, even though the stored binder is more viscous at low shear rates, its viscosity values are similar to the fresh sample above 25 s^–1^ (Figure 4B).

FTIR can be used to explain these results (Figure 5), according to Scheme 1, keeping in mind that order of addition 2 (order 2) would initially allow MDI to chemically modify the biobinder for a longer time, avoiding a prolonged processing time. The successful reaction between the NCO groups and available −OH groups was verified by the appearance of spectral bands associated with the carbonyl bond stretch of urethane units near 1700 cm^–1^.^39^ This peak appears as a shoulder of the main peak located at ca. 1730 cm^–1^ that can be associated with C=O stretching of esters, ketones, and aldehydes.^40^ According to Clemitson,^41^ the existence of the peak at 1730 cm^–1^ is a non-hydrogen-bonded carbonyl urethane group −C=O, where the peak around 1700 cm^–1^ is a hydrogen-bonded carbonyl urethane group. Comparing both addition orders, a more pronounced shoulder is observed for order 2, suggesting a higher intensity for this band and, therefore, a more extended OH/NCO reaction when processed following order 2. Furthermore, the shoulder intensity slightly decreases after 24 h of processing. In addition, it was possible to identify a spectral band associated with stretching vibrations of the O–H and N–H bonds near 3500–3200. This wide band clearly increases in intensity after 24h for order 2, suggesting that reactions continue further during hot storage.^40^

**Figure 5:**
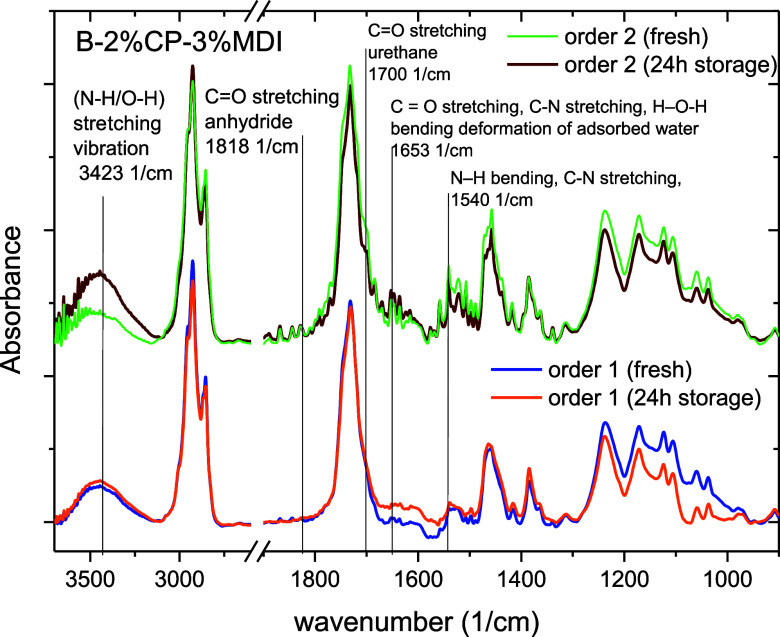
FTIR spectra of formulation B–2% CP–3% MDI, as a function of addition order, measured after sample preparation (fresh) and 24 h of storage.

Additionally, isocyanate functional groups also react with carboxylic acid groups according to two main mechanisms leading to amide and biuret products.^42,43^ The increase in the relative intensity of the band at 1653 cm^–1^ can be attributed to the contribution of the stretching vibrations of carbonyl and C–N groups of amide and urea products.^40^ Although this band may overlap with the bending deformation of adsorbed water in the coatings, which generally occurs around 1640 cm^–1^. Likewise, the band at 1540 cm^–1^ may be associated with in-plane bending deformation of N–H and stretching of C–N of amides.^44,45^

For the sake of comparison, Table 3 quantifies changes in peak intensities of the above bands for both addition orders and after a 24 h storage. Comparing spectra and relative intensities, order 2 shows all above bands with the highest intensity, suggesting that MDI addition to the rosin/oil blends initially promotes reactions with carboxylic groups, leading to amides during processing. Then, when hydroxyl-reach CP is subsequently added, order 2 also allows the formation of urethane linkages that contribute to the final polymer network. Therefore, if order 2 is selected, such a polymeric network would result in a highly cross-linked microstructure based on urethane and amide linkages. Conversely, order 1 seems to promote OH/NCO and COOH/NCO reactions to a much lesser extent than order 2, probably due to the shorter reaction time when MDI is added after CP. Although both reactions seem to continue during hot storage, with I_24h_/I_fresh_ ratio above 1 (particularly for amide bands), the final relative intensities remain far below those measured for order 2 after 24 h storage. This fact would suggest that the microstructure resulting from order 1 would have less density of chemical cross-links (i.e., more physical entanglements) than that from order 2. Interestingly, although some of the linkages are lost during hot storage for order 2, as may be deduced from the I_24h_/I_fresh_ values around 1 or below (Table 3), microstructure remains highly cross-linked. The loss in chemical linkages would also be confirmed by the increase in −OH suggested by the rise in relative intensity observed for the wide band at 3500–3200 cm^–1^, with I_24h_/I_fresh_ = 1.14 (Table 3).

**Table 3:** Relative Intensities of Bands 3500-3200, 1700, 1653, and 1450 cm^–1^ with Respect to the C–H Stretch Band 2925 cm^–1^, and Ratios between Fresh and 24 h Stored Intensities (I_24h_/I_fresh_)

	reaction: NCO/OH	reaction: NCO/COOH		OH/NH groups
	I_1700/2925_	I_1653/2925_	I_1540/2925_	I_3500–3200/2925_
order 1 (fresh)	0.221	0.017	0.058	0.133
order 1 (24 h)	0.251	0.078	0.078	0.170
I_24h_/I_fresh_	1.13	4.75	1.35	1.28
order 2 (fresh)	0.669	0.503	0.581	0.472
order 2 (24 h)	0.594	0.508	0.534	0.541
I_24h_/I_fresh_	0.89	1.01	0.92	1.14

The different polymer networks formed are shown in Figure 6. The addition order 1 leads to a fibrillar microstructure of the CP with a larger size than order 2 (Figure 6A). Likely due to ord1 being less efficient in promoting reactions between NCO and OH/COOH groups available in CP and R/O (i.e., microstructure is mainly based on physical entanglements). Conversely, order 2 that involves NCO/COOH/OH reactions to a greater extent seems to better compatibilize the CP and R/O blend with a well-dispersed polymer network of smaller fibrillar size (Figure 6C). Upon hot storage, both networks appear less structured (Figure 6B,D), but this change is more evident for the microstructure resulting from order 1 (Figure 6A,B). This result would be in good agreement with the binder softening described above, shown in Table 2, and with the less shear-thinning character shown by the 24 h storage sample processed following order 1 (Figure 4A), compared with order 2 (Figure 4B).

**Figure 6:**
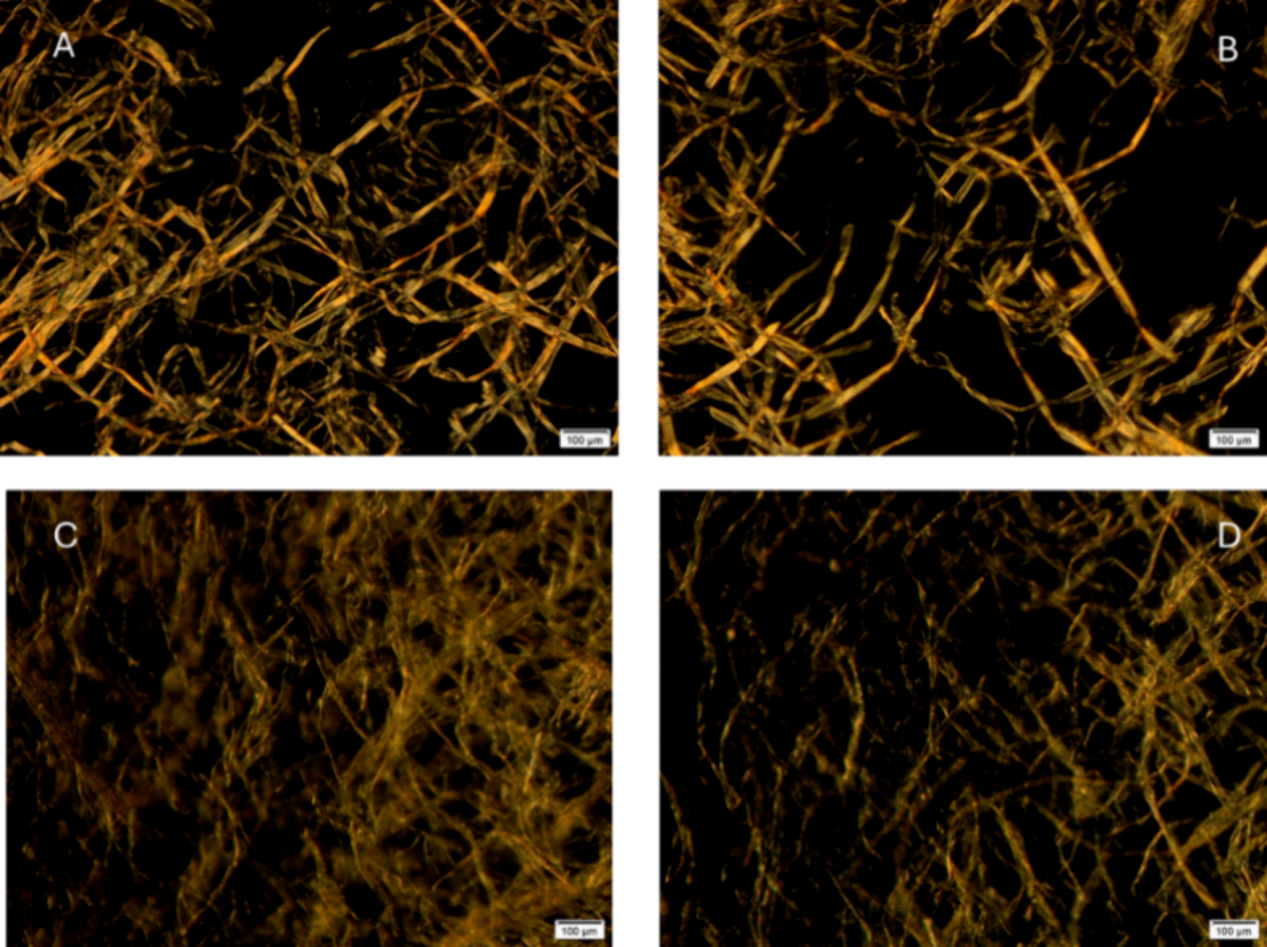
Optical polarized micrographs of (A) fresh binder processed following order 1; (B) 24-h stored binder processed following order 1; (C) fresh binder processed following order 2; and (D) 24-h stored binder processed following order 2. The bar size is 100 μm.

### Prototype Selection

Accordingly, formulation composed of 27.22 wt % oil, 67.76 wt % rosin, 2.02 wt % cellulosic pulp, and 3% MDI, processed at 150 °C following the order of addition 2, was selected as a prototype able to replace bituminous binders in road applications. Aiming to validate this assumption, the selected formulation was scaled up to a 4 kg batch using a more efficient mixing tool (a Silverson Duplex assembly). Then, the performance of the resultant binder was assessed according to the European (EN) standards for bituminous asphalt binders. As may be seen in Table 2, the processed 4 kg sample shows the values of penetration and softening point are consistent with previous preparations, no matter the batch scale, mixing device, and storage conditions considered.

According to EN standards, binder properties have to be tested on fresh samples and after being subjected to a short-term aging procedure (in a rolling thin-film oven, RTFO), which simulates binder oxidation or aging when mixed with asphalt aggregates. As demanded by the standard EN 13924–2, after aging treatment, biobinder underwent a weight loss below 0.5%, an increase in softening point below 10 °C, and retained more than 50% of its original (fresh sample) penetration (Table 4). Interestingly, if only penetration and softening point are considered, Table 4 shows that binder B–2% CP–3% MDI–ord2–4 kg would be within the category MG 50/70–54/64 applicable to multigrade bitumens listed in the standard EN 13924-2, which establishes a penetration and softening point in the ranges 50–70 dmm and 54–64 °C, respectively. Other polymer-modified bitumen (PMB) categories, gathered by the European standard EN 14023 (e.g., PMB45/8–60), could be considered, but these biobinders did not exhibit elastic recovery, a characteristic that is required for polymer-modified binders.

**Table 4:** Performance of B–2% CP–3% MDI–ord2-4 kg According to EN Standards

system	penetration (dmm)	*T*_R&B_ (°C)	after RTFOT		
			weight loss (≤0.5%)^a^ ^a^	Δ*T*_R&B_(≤+10 °C)^a^	retained penetration (≥50%)^a^
fresh binder	59	57.2	0.105%	+1.6 °C	59%
aged binder (RTFOT)	35	58.8			

a Limits set out in EN 13924-2.

Furthermore, binder performance can be assessed over a wider temperature range, from low to high in-service conditions, by using complementary rheological testing. To that end, linear viscoelastic frequency sweep tests were conducted at temperatures between −20 and 80 °C on biobinders and a neat bitumen B50/70, for the sake of comparison. Resultant frequency sweeps are presented as Black diagrams (i.e., as plots of phase angle, δ, vs complex modulus, G*) in the inset of Figure 7A. All experimental frequency sweeps approximately fall onto unique curves for every binder tested. Thus, neat bitumen shows the well-known behavior of increasing phase angle (up to 90°) as temperature rises (or complex modulus decreases), indicating continuous loss of material elastic character. Conversely, both fresh and aged biobinders show a maximum in the phase angle, increasing material elasticity at high in-service temperatures, above 40 °C, which could prevent eventual road distresses as rutting.^46^

**Figure 7:**
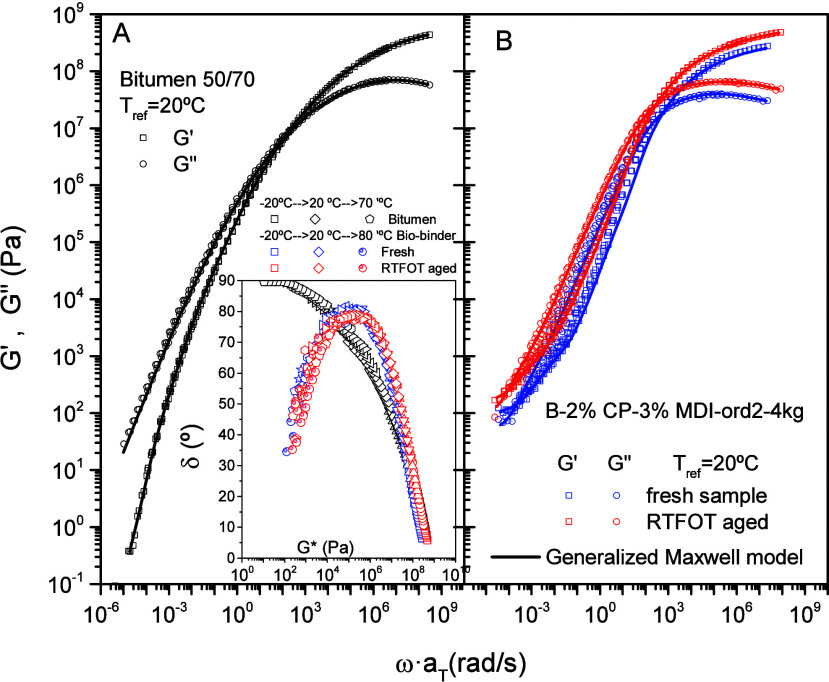
Bitumen (A) and biobinder (B) master curves of the elastic and viscous moduli at the reference temperature of 20 °C. Inset: Black diagrams applied to bitumen and biobinders.

Furthermore, black diagrams suggest that bitumen and both fresh and aged biobinders behave as thermorheologically simple materials.^47^ Using the rheological time–temperature superposition (TTSP) principle, the above frequency sweeps can be superimposed on a master curve. This procedure allows the study of material viscoelasticity over a much wider frequency range than is possible experimentally. TTSP was used to obtain the master curves of the elastic (*G*′) and viscous (*G*″) moduli at a reference temperature of 20 °C. This was achieved by means of a frequency shift factor, *a_T_*, which resulted in a reduced frequency, ω·*a_T_* (Figure 7).

Viscoelastic behavior of the reference bitumen shows a continuous transition from the glassy, with storage modulus values (*G*′) close to 10^9^ Pa, to the Newtonian region at the lowest frequencies, characterized by *G*″ values proportional to ω^1^ and *G*′ nearly proportional to ω^2^.^48^ This viscoelastic response points out the relatively low molecular weight of bitumen compounds, which are not able to form entanglements among molecules, related to the presence of a viscoelastic plateau region at low-intermediate frequencies.^49^ Fresh and aged biobinders also show the trend to reach the viscoelastic glassy region, with a high-frequency crossover between elastic and viscous moduli, which appears at a lower frequency for the aged binder. Below this crossover, both biobinders show a transition region with a predominantly viscous character (*G*″ > *G*′) toward a low-frequency crossover and, eventually, the trend to a viscoelastic plateau in *G*′. This low-frequency plateau would arise from the above-observed fibrillar network (Figure 6), formed by the high-molecular-weight (CP/MDI) polymer-rich phase.

Figure 8A shows that the shift factors, *a_T_*, decrease with temperature according to an Arrhenius-like dependence:

$$a_{T}=\exp\left[\frac{E_{a}}{R}\left(\frac{1}{T}-\frac{1}{T_{o}}\right)\right]$$ 1

where *R* is the universal gas constant, *E*_a_ is the viscoelastic activation energy, and 20 °C is the selected reference temperature (*T*_o_ = 298.15 K). Resulting activation energy values are gathered in Figure 8A and show a lower thermal susceptibility for the biobinder (*E*_a_ = 155 kJ/mol), compared with the reference bitumen (*E*_a_ = 200 kJ/mol). However, after the aging treatment, binder temperature dependence seems to slightly increase (*E*_a_ = 173 kJ/mol).

**Figure 8:**
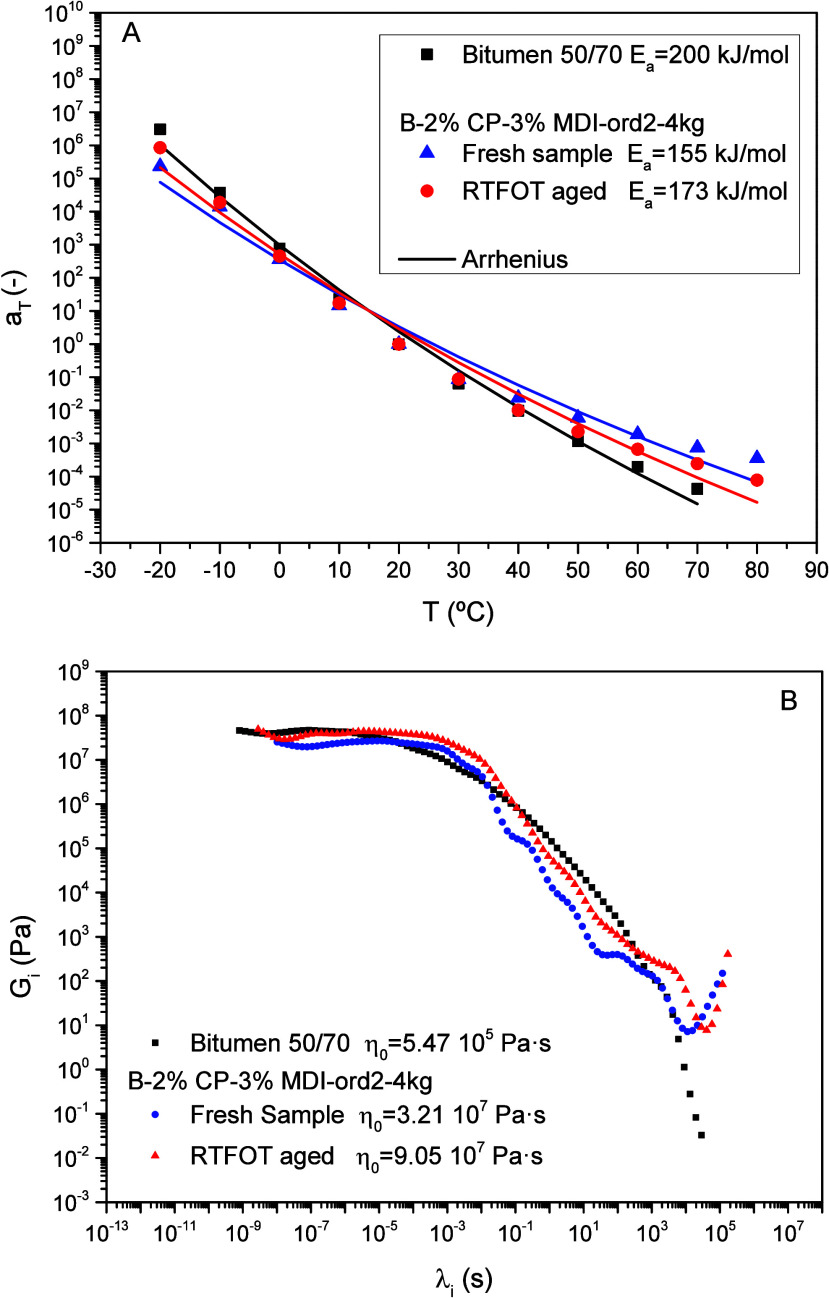
(A) Evolution of shift factor with temperature and (B) relaxation spectra calculated by fitting generalized Maxwell model (eqs 2 and 3).

Furthermore, the dynamic linear viscoelastic behavior of these systems may be described by the generalized Maxwell model:

$$G^{\prime }=G_{e}+\overset{N}{\underset{i=1}{\sum }}G_{i}\frac{{\left(\omega \lambda_{i}\right)}^{2}}{1+{\left(\omega \lambda_{i}\right)}^{2}}$$ 2

$$G^{\prime \prime }=\overset{N}{\underset{i=1}{\sum }}G_{i}\frac{\omega \lambda_{i}}{1+{\left(\omega \lambda_{i}\right)}^{2}}$$ 3

where *G_e_* is the elastic modulus, λ_*i*_ is the relaxation time, *G_i_* is the relaxation strength, and *N* is the number of relaxation times. Fittings were carried out with RheoCompass software, using *N* = 90 relaxation times for all the materials. The obtained binder relaxation spectra are shown in Figure 8B. Unlike neat bitumen, which displays a plateau at low relaxation times followed by a continuous decline, biobinder spectra show the same plateau along with a minimum at high relaxation times. The plateau at low relaxation times has been related to the resin/oil fraction,^12^ which seems to experience a hardening after aging treatment (i.e., the plateau extends to longer relaxation times with higher *G_i_* values). Finally, the minimum observed at high relaxation times would result from the nonthermoplastic biopolymer network formed by CP/MDI.

Relaxation spectra were used to obtain the low shear-limiting viscosity (η_o_) of all binders as follows:

$$\eta_{o}=\sum_{i=1}^{N}G_{i}\lambda_{i}$$ 4

The binders zero-shear limiting viscosity at the reference temperature of 20 °C is gathered in Figure 8B. Neat bitumen viscosity was around 2 orders of magnitude lower than those of the biobinders, having the aged binder the highest viscosity due to oxidation processes undergone during RTFOT.

A comparison of the material performance over a wide range of in-service conditions can also be carried out by analyzing complex modulus values (G*) at 10 rad/s as a function of temperature (Figure 9A). When comparing fresh materials (neat bitumen and unaged biobinder), biobinder exhibits a better low-temperature performance than bitumen. In this temperature region, its complex modulus values remain below bitumen and well below the viscoelastic glassy region (at G* ≈ 10^9^ Pa), where the material exhibits a lack of flexibility (i.e., becomes brittle). Biobinder oxidation is mainly observed in the low temperature region, where the increase in G* would suggest a reduced material flexibility (but with modulus values still below the glassy region). Comparing short- and long-term aging, Figure 9A shows that RTFOT treatment mostly contributes to the modulus increase, whereas RTFOT+PAV behavior would result from a balance between the oxidation of the continuous R/O matrix and the degradation of the polymer network originally formed. As forecast, the complex shear modulus decreases with the rising testing temperatures, but the biobinder curve slopes tend to level off above 40 °C, showing similar values for both fresh and aged biobinders but much higher than bitumen.

**Figure 9:**
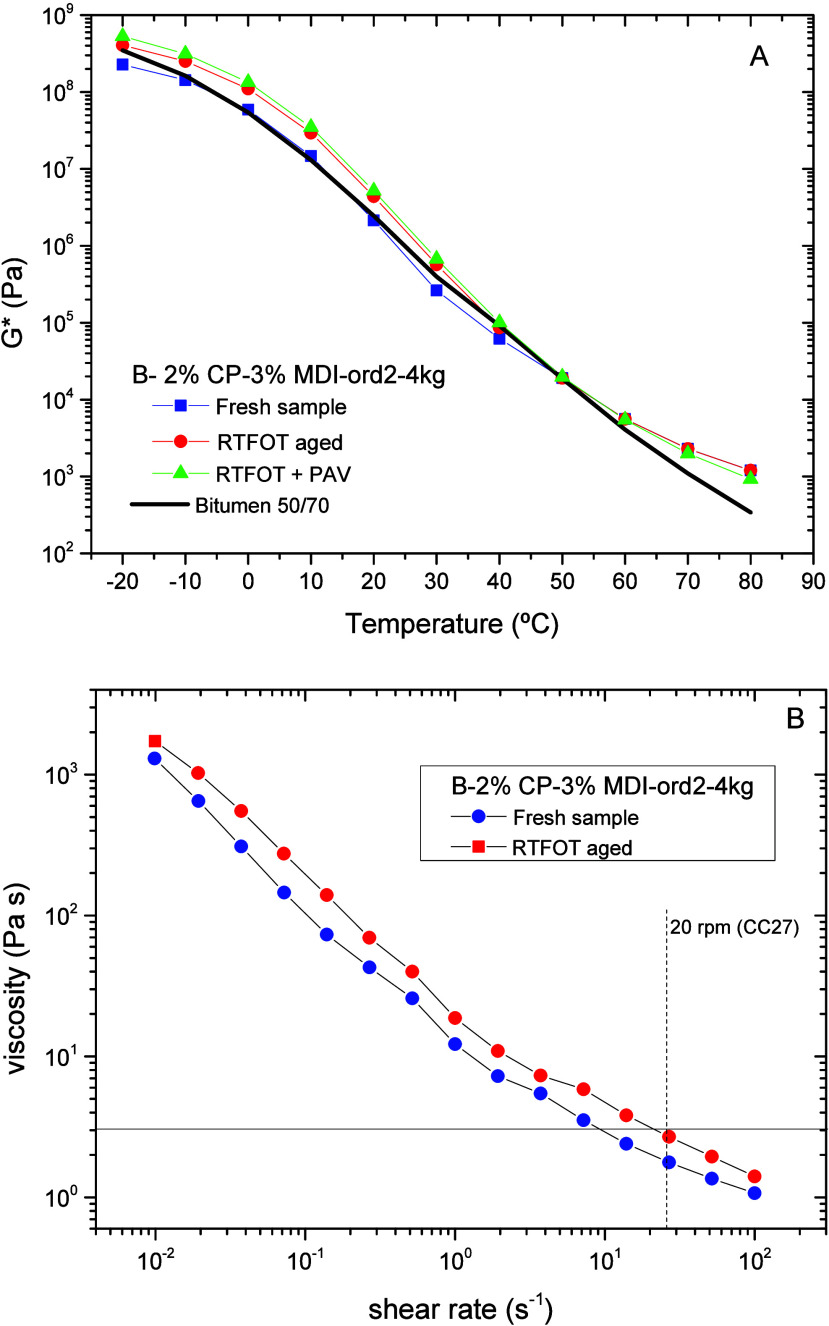
(A) Temperature dependence of the shear complex modulus (G*) of fresh and aged biobinders compared with a 50/70 bitumen. (B) Compared viscous behavior before and after biobinder RTFOT aging.

As an alternative to European standards, the American standard AASHTO MP320 proposes viscous and viscoelastic tests to set the so-called binder performance grade (PG), which defines the service temperature range of the asphalt product (i.e., the minimum and maximum pavement design temperatures). Accordingly, Figure 9B shows that biobinder viscosity would remain below 3 Pa s at shear rates above the one stablished by the standard (around 25–27 s^–1^).

Likewise, AASHTO MP320 proposed oscillatory viscoelastic tests on fresh and RTFOT-aged samples, performed in the linear viscoelastic region, to obtain the maximum pavement design temperatures (the so-called rutting parameter).^50^ This parameter is determined by recording the temperatures at which the values of G*/sin δ equal 1000 Pa for the fresh sample (*T*_1000Pa_) and 2200 Pa for the RTFOT-aged binder (*T*_2200Pa_). Such temperatures were, respectively, 84 and 73 °C (Figure 10). Although the reference SBS polymer modified bitumen PG76–28 shows a similar value of *T*_1000Pa_ ≈ 83 °C, *T*_2200Pa_ decreases less than biobinder after the RTFOT aging, down to 79 °C (giving a PG76). Accordingly, AASHTO MP320 would establish 70 °C as the maximum pavement design temperature for the selected prototype B–2% CP–3% MDI-ord2–4 kg. Interestingly, when the viscoelastic behaviors of PG76–28 and biobinder were compared after RTFOT+PAV aging, both systems present similar values for the temperatures at which the values of G*·sin δ equal 6000 kPa (*T*_6000kPa_), 17 and 19 °C, respectively. The biobinder temperature of 19 °C meets the MP320 requirements established for a PG70 after PAV aging. In this regard, it is worth noting that further tests on binders submitted to long-term aging are necessary to foresee the minimum pavement design temperature and, finally, provide the binder performance grade according to this standard.

**Figure 10:**
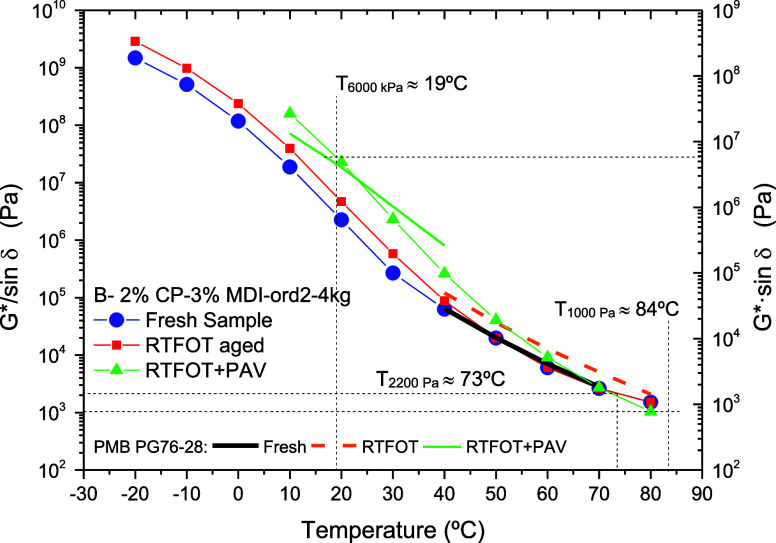
AASHTO viscoelastic characterization of biobinder B–2% CP–3% MDI–ord2–4 kg, compared with a polymer modified bitumen PG76–28.

## Conclusions

A biobased rosin ester (R), a waste cooking oil (O), and a cellulosic pulp (CP) have been used as the main components of biobinders, which should be able to fully replace petroleum bitumen as binders of aggregates in road asphalts. However, to that end, the addition of about 3% of a reactive diisocyanate prepolymer (MDI) is required. Whereas rosin is a structuring agent, oil acts as a plasticizer, and the cellulosic pulp increases material softening points, MDI plays a key role as a compatibilizer. Compatibilization takes place via urethane/amide linkages between the NCO-terminated groups of MDI and the OH/COOH groups present in the other three components (R, O, and CP). The results suggest that both amide- and urethane-based reactions occur, in that order, during processing according to order2, and that the formed network withstands 24 h storage at 150 °C better. Conversely, order 1 heavily promotes urethane linkages during processing and the amide linkages during hot storage, but both reactions take place, to a much lesser extent, if compared with order 2.

As a result, a biobinder composed of 27.22 wt % oil, 67.76 wt % rosin, 2.02 wt % cellulose pulp, and 3 wt % MDI, which was processed at 150 °C following the order of addition 2 (R > O > MDI > HPC), may be proposed to replace bituminous binders in road applications. If the European standards for asphalt binder are considered, this formulation is expected to show a performance similar to the so-called multigrade bitumens, being classified as MG 50/70-54/64 according to EN 13924-2. Likewise, if the American standards are considered, this biobinder would be initially applicable up to a maximum pavement design temperature of 70 °C (AASHTO MP320).

However, to better elucidate the industrial application of these products, other considerations such as the cost of the final product, the availability of waste, the assessment of the long-term behavior of the resulting asphalt mix, health and environmental issues arising from the release of NCO during processing/laydown, etc., as well as alternative hybrid strategies (e.g., blending with reclaimed asphalt, RAP) should be taken into account.
